# Nanotechnology in the Future Treatment of Diabetic Wounds

**DOI:** 10.1900/RDS.2020.16.1

**Published:** 2020-07-01

**Authors:** Robert A. Smith

**Affiliations:** University College London, Medical School, London, United Kingdom.

**Keywords:** diabetes, nanotechnology, wound healing

## Abstract

Diabetic wounds have a large and increasing burden on the healthcare of the UK. Currently, none of the standard treatment options for the treatment of diabetic wounds specifically target the physiological processes behind their enhanced severity. This review evaluated recent studies in the field of nanotechnology concerned with treating diabetic wounds. The studies had each developed novel therapeutics involving nanomedicines that sought to either enhance angiogenesis, the construction of new blood vessels, or increase collagen production, as well as limit the augmented inflammation, in wounds in diabetic rat or mice models. The investigations tended to either target specific anti-inflammatory or pro-proliferative receptors on endogenous cells, or transport growth factors to the wound. Previous studies have shown the beneficial effects of growth factors on healing, but they are easily broken down. By transporting them in nanoscaffolds and liposomes, it has been shown that the longevity of growth factors can be enhanced. Gold nanoparticle matrices have also been shown to have a beneficial effect on healing, by both conveying proliferative factors and independently triggering angiogenesis and collagen production. The most impressive results in the review were achieved by nanomedicines involving multiple growth factors, hence, the review will highlight the beneficial factors to wound healing and suggest a composite therapy to be trialled in the future. The review will evaluate each set of papers using similar nanomedicines and highlight the challenges of transferring this therapy to the clinic.

## 1 Introduction

Whilst diabetes is generally associated with diet and the gastrointestinal system, the disease also has a detrimental impact on wound healing, with significant clinical consequences if left untreated. Diabetic wounds are characterised by excessive inflammation, which damages healthy tissue and prevents an effective immune response and healing process [1]. Patients with diabetes are also more likely to suffer from an open wound in their lower extremities due to the peripheral neuropathy associated with diabetes [1]. Together, these factors increase the probability of infection, gangrene and amputation in diabetic patients [1].

The health care cost of diabetes is estimated to be $116 billion in the US, with over a quarter of that figure spent on the treatment of chronic diabetic wounds [2]. Furthermore, in the UK, lower limb amputations and aftercare costs almost 1% of the NHS (National Health Service) budget [3]. This problem is likely to intensify, with the Center for Disease Control and Prevention predicting that a third of the US adult population will have contracted diabetes by 2050 [4].

Given that the problems associated with diabetic wounds are so ubiquitous, it is surprising that NHS standard practice for treating diabetic wounds is non-specific; therapy involves cleaning and monitoring the wound [5]. Therefore, the clinical applications of nano-therapy that directly targets the defective healing processes associated with the diabetic wound could be widespread. The following review will seek to critically appraise the various studies in this field and evaluate their clinical applications. The most recent studies in the field will be evaluated, with an emphasis on 3 different therapeutics: Nanofiber scaffolds, gold nanoparticles and liposomes.

To fully outline the mechanisms and limitations of nanotherapy in diabetic wound healing, first a physiological and pathological context must be established concerning inflammation. Inflammation is an immediate and innate response to tissue damage and is usually not a prolonged state [6]. However, in diseases such as diabetes, inflammation can become a chronic phase [6]. This occurs mainly due to the hypoxia in the wound, induced by the high oxidative stress of a glucose rich environment [7]. This hypoxia has two critical effects: it reduces angiogenesis (the formation of new blood vessels) and it reduces the expression of multiple growth factors, thereby preventing the formation of a stable collagen matrix [7]. It is worth noting that whilst the formation of a stable collagen matrix requires multiple factors, such as FGF (fibro-blast growth factor), EGF (epidermal growth factor) and PDGF (platelet-derived growth factor) amongst others, the process of angiogenesis is pinned upon the expression of VEGF (vascular endothelial growth factor) [6]. The vast majority of the investigations that are evaluated in this review have therefore looked at upregulating either angiogenesis, by targeting compounds in the VEGF pathway or by applying VEGF itself to the wounds directly, or collagen formation, by attempting to deliver growth factors to the wound or upregulating fibroblast proliferation, the key cell required to move the healing process beyond the inflammatory phase [8].

## 2 Methods

A review of the literature was conducted to identify published primary research articles relating to nanotechnology and diabetic wound healing. The following search strategy was applied on PubMed with no date or language restrictions:

(Nano*) AND (Diabet*) AND (Wound OR Injury OR Heal*)

Articles were screened by title followed by abstract and by full review. Finally, reference lists of included documents were used to identify additional eligible documents (forward and backward citation tracking).

Documents were eligible for inclusion if they clearly involved primary research concerning the application of a nanotherapy in wound healing in a diabetic model.

## 3 Results

### 3.1 Overview

Table A1 provides an overview of the therapeutics, results and conclusion of the reviewed studies. Whilst many of the studies included *in vitro* investigations into the mechanics, dynamics and toxicity of their various therapeutics, emphasis has been made, on evaluating *in vivo* trials, thereby rendering the studies more comparable and clinically relevant. Where *in vitro* findings were particularly relevant, they have been included in detail below.

### 3.2 Main results

All of the *in vivo* studies reviewed started by inducing diabetes in either rats or mice. This was achieved in most of the studies by injecting a toxin to insulin producing cells of the pancreas, such as streptozotocin [14], and also a citrate compound, to quickly induce hyperglycaemia in tissue. The blood glucose levels were then monitored, to determine whether diabetes had been successfully induced in the animals. This approach has its limitations though, as the complex deficiencies of the diabetic wound are not induced immediately [7], and therefore the mere weeks in which the animals were converted from an insulin competent to insulin deficient state, may not be long enough to truly mimic the defective physiology of the diabetic wound. It is also notable that for each study involving animals, less than 10 were involved in each treatment line, thereby increasing the probability of anomalous disruption to the investigations.

In most cases the diabetic rats were then surgically wounded. Most of the wounds were around 1cm in diameter, with the largest being 2cm [12], [20], [29] and the smallest being 5mm [26]. The wounds were then treated with their respective nanomedicines and control solutions, these were mostly applied as a dressing or within a surgical gauze. A measurement relating to wound area was then taken across a period of around two weeks after the initial injury, with the shortest trial length being 7 days [27], [28] and the longest being 28 days [30]. This similarity in procedure means that the various trials are relatively comparable and therefore this review will seek to draw common conclusions from them.

Whilst all the trials involving nanofibers sought to create a nanoscaffold structure conducive to healing, there was no common target or mechanism. One study [9], sought to partially activate the avβ3 integrin on endothelial cells, thereby leading to increased expression of intercellular adhesion molecule 1 (ICAM-1) and increased angio-genesis. The activation motif of this particular integrin was a triplet of amino acids: arginine, glycine and aspartate (RGD) [9]. The peptide nanofibers under investigation in the study contained a motif of Arginine, alanine and aspartate (RAD), hence the interaction between the medicinal substance and the endothelial cells was indirect. However, this investigation showed that this relationship was ideal for upregulating ICAM-1 expression; the RGD containing control was less effective at inducing angiogenesis, and by extension wound healing, than the RAD nanofibers [9]. Of all the studies reviewed, this mechanism seemed the most complex, and therefore in many ways the most problematic, as it could be argued that it would be unlikely to transfer from temporarily diabetic mice, with small wounds, to permanently diabetic humans, with larger injuries.

Most of the other nanofiber trials were more direct in their approach. They sought to transfer growth factors, beneficial to wound healing, straight to the site of injury. When applied alone to a wound, growth factors are rapidly broken down. However, nanoscaffolds and other nanotherapies may be able to provide a protective casing for growth factors, thereby enabling them to be applied without degradation [32].

Multiple studies involved a nanoscaffold-containing epidermal growth factor (EGF) [11, 21, 23, 24]. EGF has a beneficial impact on angiogenesis, granulation and contracture within a wound [33]. For some this was the major therapeutic factor of the study [11, 21], whilst for others, it was a co-factor, used in conjunction with other therapeutics in a bid to greatly augment the healing process [23, 24]. One study used it in company with the antibacterial compound gentamicin sulfate [24], whilst the other combined it with silk fibroin from silkworms, and used species of silkworm as a dependent variable [23]. Whilst three of the studies concluded that EGF was important in the early stages of wound healing [11, 23, 24], one concluded that it was a more significant factor in later stages [21]. This disparity may be due to the outlying study [21] using an EGF-containing control and also a second therapeutically significant compound, poly (lactic-co-glycolic acid) (PLGA). The PLGA may have prolonged the EGF action and the EGF containing control would have accentuated the therapeutic effects of the carrier system, rather than the EGF itself, in this study. Therefore, in balance, it can be concluded that EGF is more important in the early stages of diabetic wound healing. This may be explained by the close relationship between EGF and angiogenesis, an important constituent process of the early stages of wound healing [34].

A second growth factor that was investigated by multiple studies was PDGF (platelet derived growth factor) [13, 19, 26]. Whilst EGF targets the process of angiogenesis, by upregulating endothelial cells proliferation, PDGF is more important in the proliferation of fibroblasts and therefore collagen production [34]. One study was conducted *in vitro* and was able to reinforce the strong link between fibroblast proliferation and PDGF, whilst also ascertaining that PDGF applied via a nano-scaffold has a more prolonged length of activity than PDGF applied on its own [13]. The other two studies involving PDGF both involved *in vivo* investigations [19, 26]. The two studies demonstrated that the positive effects of PDGF lasted for the entirety of the healing process, with the active nanomedicine group inducing wound healing at all stages of the two investigations [19, 26]. Although these studies were small in sample size, and therefore the conclusions were made with relatively high p-values (p < 0.05 in both cases), the role of PDGF, applied by nanofiber scaffolds, looks to be universally beneficial to diabetic wound healing at this stage.

Two of the other nanofiber studies under review directly involved growth factors, these were G-CSF [12] and VEGF/FGF (fibroblast growth factor) [25]. G-CSF has a beneficial effect on most stages of wound healing, including both collagen production and angiogenesis [35]. The G-CSF study was able to conclude that its beneficial effects of fibroblasts were present throughout the *in vivo* healing process after being applied via a nanoscaffold and that, for the initial stages (14 days), this translated into increased wound healing [12]. VEGF has a direct effect on angiogenesis [6], whilst FGF is vital for fibroblast proliferation and therefore collagen production [25]. The study was able to conclude the VEGF/FGF composite had a beneficial effect on wound healing, however there was no point in the investigation in which the nanoscaffold was more effective than the growth factor scaffold control [25]. Furthermore, not only was the growth factor scaffold control slightly more effective than the nanomedicine (albeit not statistically significantly), the difference between the therapeutics and the complete control groups was only slight [25]. This may be due to the differences in growth factor release between the nanoscaffold and the growth factor scaffold: the nanoparticles may have diffused across the fibrin scaffold and therefore broken down more quickly [25]. The growth factor scaffold, meanwhile, may have protected the growth factors more effectively until their optimal window of action [25].

Two of the other nanofiber studies under review directly involved growth factors, these were G-CSF [12] and VEGF/FGF (fibroblast growth factor) [25]. G-CSF has a beneficial effect on most stages of wound healing, including both collagen production and angiogenesis [35]. The G-CSF study was able to conclude that its beneficial effects of fibroblasts were present throughout the *in vivo* healing process after being applied via a nanoscaffold and that, for the initial stages (14 days), this translated into increased wound healing [12]. VEGF has a direct effect on angiogenesis [6], whilst FGF is vital for fibroblast proliferation and therefore collagen production [25]. The study was able to conclude the VEGF/FGF composite had a beneficial effect on wound healing, however there was no point in the investigation in which the nanoscaffold was more effective than the growth factor scaffold control [25]. Furthermore, not only was the growth factor scaffold control slightly more effective than the nanomedicine (albeit not statistically significantly), the difference between the therapeutics and the complete control groups was only slight [25]. This may be due to the differences in growth factor release between the nanoscaffold and the growth factor scaffold: the nanoparticles may have diffused across the fibrin scaffold and therefore broken down more quickly [25]. The growth factor scaffold, meanwhile, may have protected the growth factors more effectively until their optimal window of action [25].

Multiple studies in the review also investigated the role of curcumin in diabetic wound healing [16], [17], [18]. This molecular component of turmeric, the Indian spice, has been shown to have anti-tumour properties [36]. However, its anti-cancer mechanism has been suggested to involve anti-angiogenesis, therefore it could be assumed to have a detrimental effect on wound healing [36]. This is in direct contradiction to some of the studies reviewed here; it was shown to significantly increase wound healing in these cases [16], [18]. This may be due curcumin’s positive effects on fibro-blast proliferation, and therefore collagen production [16], and also its anti-inflammatory properties [18]. However, one study was unable to conclude, with statistical significance, that curcumin was beneficial for wound healing [17]. This could be explained by its supposed conflicted mechanism: on the one hand it increases collagen production [16], [18], whilst on the other, it inhibits angio-genesis [36]. At this stage, it is difficult to conclude absolutely whether a curcumin loaded nanoscaffold is beneficial to wound healing or not.

Multiple studies assessed the efficacy of a metformin, also known as glucophage, containing nanoscaffold on diabetic wound healing [10, 14]. Metformin is an anti-hyperglycaemic compound, which has been shown to reduce liver glucose production [37]. The studies both combined metformin with PLGA and created a nanofiber matrix via electrospinning [10], whilst one also applied collagen itself to the matric under investigation [14]. Both studies were able to conclude that a metformin containing nanoscaffold is beneficial to wound healing, yet the results from the study that included collagen were no more statistically significant than those from the study that omitted it, in fact, they were not as statistically strong (the p-value was higher in the case of the collagen trial) [10, 14]. The studies were similar in design: they both measured wound healing for 14 days and the size of the diabetic wound was 8mm in diameter in all cases [10, 14]. However, it should be noted that whilst the collagen free trial used mice, the collagen trial used rats [10, 14]. This suggests that the anti-hyperglycaemic properties of metformin are beneficial to diabetic wound healing, yet the addition of collagen to a nanofiber matrix may be superfluous. Yet, it should be noted that another trial included a component of collagen, componential collagen polycaprolactone (PCL), in its nano-scaffold [20]. This trial demonstrated an increased rate of angiogenesis, by measuring CD31 (endothelial cell) count, in wounds to which its matrix had been applied, with a reasonable level of certainty (p<0.05) [20]. However, the nanoscaffold in this trial also included other bioactive components (glass nanoparticles), so it is difficult to determine the extent of the action of the collagen component in the increased wound healing [20]. Moreover, the collagen used was only a collagen component and not the full molecule [20]. Hence, it is difficult to conclude that there is a direct link between the application of collagen to a diabetic wound therapeutically, and increased wound healing.

Two studies in the review evaluated the use of silicon ions in the treatment of diabetic wounds [15], [29]. The use of silicon has long been shown to improve patient outcome in terms of wound healing, but few trials have considered the uses of nanotechnology in improving its application [39]. However, their approaches were incomparable, one used a nanoscaffold containing silicon ions [15], whilst the other combined silica ions with gold nanoparticles [29]. The silicon nanoscaffold study was able to conclude with great certainty (p < 0.001) that silicon ions were conducive for greater angiogenesis (measured by CD31 cell count) and wound healing [15]. The gold trial was able to come to a similar conclusion in terms of wound healing, but was also able to determine that that the gold silicon composite upregulated fibroblasts and therefore collagen production [29].

The final trial involving a nanoscaffold investigated the link between upregulation of the transcription factor, hypoxia inducible factor 1 alpha (Hif-1α), and increased angiogenesis [22]. Hif-1α is a ubiquitous transcription factor that is involved in stoke, cancer, erythropoiesis and energy metabolism [39]. The trial was able to conclude that upregulation Hif-1α resulted in increased angio-genesis and faster diabetic wound healing [22]. The nanoscaffold used in the study included desferrioxamine (DFO), which alongside acting on Hif-1α is also an iron chelator. The chelation of Fe2+ ions in the wound reduced oxidative stress and therefore removed some of the impediments of healing specific to diabetic wounds [22].

Aside from the previously discussed gold, silicon composite trial [29], two other investigations looked at the effects of gold nanoparticles on diabetic wound healing [27, 28]. Both trials used similar materials in their therapeutics: epigallocate-chin gallate (EGCG) and alpha lipoic acid (ALA), alongside gold nanoparticles [27, 28]. The tea extract EGCG has been shown to reduce inflammation and also downregulate telomere erosion [40]. The two studies were both able to conclude that diabetic wound healing was increased by their therapeutic matrix, with both the anti-inflammatory properties of EGCG and the endothelial proliferative advantages of gold nanoparticles theorized to be responsible [27, 28]. The second study used a gas carrier, N2, when applying therapeutics to the wounds [28]. This was said to improve distribution and activity of the therapeutics under investigation [28].

The final two trials under investigation both used liposomes as their nanomedicine and there was a great disparity in their results. The first trial involved the pro-angiogenic factor stromal cell derived factor 1 (SDF-1) [30]. This growth factor was combined into liposomes and then applied to diabetic wounds [30]. However, the study was only able to conclude, with limited certainty (p < 0.05), that wound healing was improved in the nanoparticle group for part of the duration of the investigation [30]. For most of the study there was no difference in healing between the medicine and the control groups [30]. The second study involved a composite of 3 different growth factors, all combined with liposomes (PDGF, EGF and IGF (insulin growth factor)) [31]. This study was able to conclude with a great level of certainty (p < 0.001) that the liposome composite induced a greater healing rate in diabetic wounds [31]. This suggests that an agglomeration of various growth factors in a liposomal arrangement, or nanoscaffold, may be a particularly effective therapeutic strategy for treating diabetic wounds.

## 4 Conclusions

The overwhelming positive results throughout the review demonstrate that the field of nanotechnology has many potential applications in the treatment of diabetic wounds. It is also worth noting that these results are particularly encouraging in terms of their clinical applications, especially given that there are currently no specialised NICE (National Institute for Health and Care Excellence) approved standard treatments for diabetic wounds [5]. However, these trials do not definitively prove the efficacy of the various nanomedicines they evaluated, for although most were undertaken *in vivo*, the sample size for each treatment group was small and few of the studies were directly comparable. Furthermore, there is a key difference in wound healing between rodents and humans: rodents have looser skin which renders wound contracture more prominent in healing than in humans [41]. Therefore, as rodent wounds heal faster naturally, positive results from the studies in this review are likely to be less pronounced if repeated on human subjects.

Moreover, there was very little consensus in the studies about the best constituents for a nanoscaf-fold or liposomal matrix to treat diabetic wounds. Various growth factors were trialled, as well as more targeted medicines that sought to trigger a certain receptor. I would like, therefore, at this point to suggest a possible combination therapy for future trials based on this review. Although the results for gold nanoparticles were positive, there are excretory and financial concerns about their clinical viability [42]. It was clear that the most efficacious therapies involved multiple growth factors [31], therefore a future trial should involve a nanoscaffold, or liposomal matrix, that contains more than one active compound. It was also concluded that EGF had advantages in the early stages of healing [11], [23, 24], therefore it would be prudent to combine it with a factor that was more active later in the process, such as curcumin [16]. Factors such as silicon ions, PDGF and DFO were also shown to enhance healing, but via different mechanisms. Furthermore, compounds conducive to collagen construction, such as hyaluronic acid, and antibacterial compounds, such as gentamicin, also had beneficial effects on healing. Therefore, I would hypothesise that a composite nanoscaffold containing EGF, PDGF, DFO, curcumin, silicon ions, hyaluronic acid and gentamicin would be particularly beneficial to diabetic wound healing, as each of these constituent compounds have been shown to improve healing in the diabetic wound by individual mechanisms. If such a therapy were to be successful in an *in vivo* model, it could accelerate the instigation of a future clinical trial.

It could be possible to combine a nanoscaffold containing growth factors into a dressing used in the treatment of a diabetic wound in clinical practice. Given the regular high frequency of contact between patients and practitioners in specialist foot clinics, a large-scale clinical trial could be feasible in the near future. However, the considerable cost of transitioning between small-scale rodent injuries and much larger human wounds remains a significant barrier to such an endeavour. Each of the growth factors included in a nanoscaffold is expensive, and a combination therapy, whilst appearing the most clinically beneficial, would also incur the greatest cost.

In summary, the results included in this review reveal a potential new avenue in the treatment of diabetic wounds. A nanoscaffold involving a combination of growth factors would appear to carry the greatest therapeutic potential, but such a therapy would incur a significant economic cost.

## Appendix

**Table A1.** An overview of the therapeutics, results and conclusions of the reviewed studies

Nanomedicine	Therapeutic	*In vitro* / *in vivo* model	Results	Conclusions	Reference
Nanofibers	RAD 16-II (amino acid sequence – Arg-Ala-Asp) peptide nanofibers mainly targets revascularisation	Both *in vivo* and *in vitro. In vitro* model: Primary microvascular endothelial cells (MVECs) isolated from mouse lung tissue. *In vivo* model: Diabetic induced mice were wounded – extent of healing of nanofibers was compared with that of saline and nanofibers with no affinity (KFE-8 (LysPhe-Glu)).	*In vitro*: formation of robust capillary like networks at 24h. *In vivo*: noticeable wound closure and granulation tissue formation at day 7 (p<0.01).	The low affinity of RAD to the RGD (Arg-Gly-Asp) motif of the integrin αvβ3 produced granulation tissue more quickly than a high affinity ligand (RGD), or a ligand with no affinity (KFE-8).	Cho H et al., 2012 [9]
Nanofibers	PLGA (poly (lactic-coglycolic acid)) and metformin were first dissolved in HFIP (hexafluoroisopropanol) and spun into nanofibrous membranes	*In vivo* model: Diabetic induced mice were given wounds 8mm in diameter. The extent of healing of PLGA/ metformin nanofibers was then compared with that of virgin PLGA and a gauze sponge.	Healing of the PLGA/ metformin nanofibers was statistically greater that the 2 control groups after 14 days (p< 0.01).	The metformin delivered by the nanofibers enabled the construction of a water-soluble matrix that was conducive to reepithelisation.	Lee CH et al., [10]
Nanofibers	EGF was ingrained (rhEGF nanofiber) and contained within (rh-EGF nanofibers) nanoscaffolds (2 separate therapies).	*In vivo* model: Wounded diabetic mice healing was measured for 14 days under 4 different therapies: rh-EGF nanofiber, rh-EGF nanofibers, nanofibers and a saline control.	Healing of the rhEGF nanofiber treatment was statistically greater than the other 3 groups after 7 days but not after 14 days (p<0.05)	EGF is required for the early stages of diabetic wound healing and a small dose of EGF ingrained in a nanoscaffold can improve healing.	Choi JS, et al., [11]
Nanofibers	G-CSF loaded chitosan nanoparticles incorporated in PCL (polycaprolactone) nanofibers, followed by surface coating with collagen type I.	*In vivo* model: Male rats were given 2 cm diameter wounds. Rats were split into 4 groups and wounds were either covered in gauze, a hydrocolloid dressing, an empty nanoscaffold or a nanoscaffold with GCSF.	The G-CSF nanoscaffold significantly reduced wound area for the first 14 days (p<0.05), with significantly more collagen and fibroblasts in the G-CSF nanoscaffold wound than the controls throughout the 21 days of the investigation.	The release of GCSF from a nanoscaffold can improve fibroblast proliferation, collagen production and reduce scarring.	Tanha S et al., 2017 [12]
Nanofibers	PLGA microspheres incorporated into PLLA (polylactic acid) nano-fibrous scaffolds.	*In vitro* model: The nanoscaffolds were added to plates of human fibroblasts and the rate of PDGF production of the fibroblasts for 45 days. The quantity of PLGA on each microsphere was the following set: {10ng / mg, 100ng /mg, 300ng /mg, 600ng / mg, 1000ng / mg}. PLGA microspheres unincorporated into nanoscaffolds and saline solution (PBS – phosphate buffered saline) were used as controls.	After 45 days the most PDGF had been produced by the fibroblasts with the scaffolds containing 300ng /mg of PLGA on each microsphere (p<0.05). PDGF production quickly tailed off in the unincorporated microspheres.	Nano-fibrous scaffolds containing PLGA microspheres can induce the production of the PDGF growth factor in fibroblasts. The incorporation of PLGA microspheres into a scaffold significantly reduced their degradation.	Wei G et al., 2006 [13]
Nanofibers	PLGA, glucophage, and collagen were dissolved in 1,1,1,3,3,3hexafluoro-2propanol and were spun into nanofibrous membranes.	*In vivo* and *in vitro* model (In vitro model to measure collagen release). In vivo model: Diabetic rats were given 8mm wounds. Wounds treated with either active nanoscaffold, empty nanoscaffold or a plain gauze.	Wound closure of the active nanoscaffold group was significantly greater than the gauze group and empty scaffold group after 7 days and greater than the gauze group after 14 days (p<0.05).	A nanoscaffold containing factors required for healthy collagen production will induce greater re-epithelisation, collagen production and wound healing in diabetic rats.	Lee CH et al., 2015 [14]
Nanofibers	Poly (caprolactone) (PCL) / gelatin nanofibrous composite scaffold containing silicatebased bioceramic particles (Ca7P2Si2O16).	*In vivo* and *in vitro* (*in vitro* used to analyse vascular generation properties of scaffold on human epithelial cells). *In vivo* model: Diabetic mice were given 8mm wounds. Wounds were treated with either PCL, PCL composite nanoscaffolds or nothing.	The CD31 count was significantly greater in the nanoscaffold group than the other two groups throughout the 15 days of the in vivo study (p<0.001). The level of TGF (alpha and beta) and IL-1 was also significantly lower within the nanoscaffold group throughout the 15 days of the study (p<0.001).	The use of silicon ions in a nanoscaffold can significantly upregulate the proliferation of cells required for helaing in a diabetic wound, as well as significantly downregulating pro-inflammatory factors.	Lv F et al., 2017 [15]
Nanofibers	Curcumin incorporated into chitosan nanoparticles and impregnated into a collagen nanoscaffold.	*In vivo* and *in vitro* (*in vitro* to measure curcumin release of scaffold.) *In vivo* model: Diabetic rats were given 400mm2 wounds. The wounds were treated with either a sterile gauze, a scaffold without curcumin or a curcumin scaffold. Inflammation was also measured during the time period by measuring heat levels.	Wound contraction in the active nanoscaffold group was significantly greater than the other 2 groups after 3 and 7 days (p<0.05) and significantly greater than the other two groups after 11 and 15 days (p<0.01). Heat flow was also reduced in the active nanoscaffold group.	Curcumin application via a nanoscaffold can enhance anti-inflammatory effects and duration, especially in the later stages of wound healing.	Karri VV et al., 2016 [16]
Nanofibers	Curcumin loadedpoly (3-hydroxy butyric acid-co-3hydroxy valeric acid) (PHBV) nanofibers (fabricated via electrospinning)	*In vitro* model: 0.1, 0.3, and 0.5 % curcumin nanofibers, saline solution and an empty nanoscaffold were all added to mouse fibroblast cells and the % of viable cells was measured after 72 hours.	There was no statistical difference in cell viability between the 5 groups after 72 hours (p<0.01), although a slight increase in cell number was observed with increasing curcumin concentration.	There is negligible cytotoxicity of curcumin laced nanoscaffolds on fibroblasts and there is a slight positive trend in viability with increasing curcumin concentration.	Mutlu G et al., 2018 [17]
Nanofibers	Curcumin-loaded poly (εcaprolactone) (PCL) / gum tragacanthin (GT) nanofibers (fabricated via electrospinning)	*In vivo* and *in vitro. In vitro* model: Nanoscaffolds containing curcumin were added to populations of the bacteria MRSA (methicillin resistant staphylococcus aureus) and ESBL (extendedspectrum beta-lactamase producing E. coli). The rate of bacteria growth was compared to a control (untreated bacter ial populations). *In vivo* model: Diabetic rats were given 10mm diameter wounds. The wounds were treated with either empty or cell lined scaffolds.	*In vivo* model: The curcumin laced nanoscaffold demonstrated antibacterial growth restrictions of 99.9% against MRSA and 85.14% against ESBL. *In vitro* model: The curcumin nanoscaffold induced significantly greater healing than the control throughout the investigation and greater healing than the acellular scaffold after 10 and 15 days (p<0.05).	The application of curcumin laced nanofibers may improve the efficacy of antimicrobial compounds as well as significantly increasing the rate of healing of the diabetic wound by regulating the release of the anti-inflammatory compound curcumin.	Ranjbar-Mohammadi M et al., 2016 [18]
Nanofibers	rhPDGF-loaded PLGA membrane laced nanofibers (fabricated via electrospinning)	*In vivo* and *in vitro* (*in vitro* model used to determine structural integrity of scaffolds). *In vivo* model: Diabetic rats were given wounds of 8mm in diameter. The wounds were treated with either rhPDGF-loaded nanofibrous membranes, PLGA only membranes or an empty gauze.	The active nanoscaffold significantly reduced the wound area compared with the other two groups throughout the investigation: at days 3, 7 and 14 (p<0.05).	A PDGF laced nanoscaffold can continue to release the pro-healing factor PDGF throughout the healing process of a diabetic wound. thereby significantly increasing the healing rate.	Lee CH et al., 2015 [19]
Nanofibers	A nanofibrous matrix composed of ECM (extracellular matrix) componential collagen polycaprolactone (PCL), and bioactive glass nanoparticles (BGNs).	*In vivo* model: Diabetic rats were given wounds of 2cm in diameter. Wounds were treated with either the active nanoscaffold or a mixture of PCL and ECM collagen or were not treated at all. The wound healing rate was measured for 21 days and the CD31 count was also measured in order to determine the rate of revascularisation.	The active nanoscaffold produced a greater rate of healing between days 4 and 21 than the other two models (p<0.05). CD31 count was also significantly greater in the nanoscaffold group throughout the investigation (p<0.05).	The nanofibrous matrix significantly enhanced cell proliferation and angiogenesis in the diabetic wound. This is most probably via the VEGF pathway.	Gao W et al., 2017 [20]
Nanofibers	rhEGF nanoparticles emulsified with poly (lactic-coglycolic acid) to create a nanoscaffold.	*In vivo* model: Diabetic rats were given a wound 1.8cm in diameter. The wounds were then treated with either rhEGF nanoparticles, an rhEGF solution, empty nanoparticles or saline solution. Healing rate (primitive area – nonhealing area / primitive area) was then calculated.	The healing rate of the rhEGF nanoparticles was significantly greater than the saline and empty nanoparticles after 7 and 14 days and also significantly greater than the rhEGF solution after 21 days (p<0.01).	A nanoscaffold containing rhEGF is a particularly efficacious method for the treatment of diabetic wounds, especially in their later, post-inflammatory stages.	Chu Y et al., 2010 [21]
Nanofibers	Desferrioxamine (DFO) added to PVA-CS (poly (vinyl alcohol) / chitosan) hydrogel nanofibrous scaffolds.	*In vitro* and *in vivo* model (the in vitro model determined the mechanism of action of DFO scaffolds). *In vivo* model: Diabetic rats were given a wound of diameter 15 mm. The wounds were treated with either DFO hydrogel scaffolds or just hydrogel solutions.	The nanoscaffold group demonstrated a significantly reduced wound area than the hydrogel group between days 6 and 18 (p<0.01).	The Fe2+ chelator DFO scaffolds upregulates the expression of Hif (Hypoxia inducible factor) 1 α, and therefore VEGF, thereby increasing the rate of revascularisation and wound healing.	Chen H et al., 22
Nanofibers	Silk fibroin (from silkworms) was added to rhEGF, PVA (poly (vinyl alcohol) and FGF and then electrospun to create a nanoscaffold.	*In vivo* model: Diabetic rabbits were given wounds 12mm in diameter. The wounds were then treated with nanoscaffolds composed of silk fibroin from 3 different species of silkworm (Antheraea assama, Bombyx mori, Philosamia ricin) as well as a nanoscaffold without silk fibroin and a saline control.	The silkworm nanoscaffolds induced a significantly greater rate of wound closure than the controls for the first 14 days, with the Bombyx mori scaffold significantly less efficacious in this period. However, by 21 days after the wounds were made, there was no significant difference in wound area between all the nano scaffold groups, although the saline control wound was still significantly behind in terms of closure (p<0.05).	The silk fibroin enhanced scaffolds may significantly improve the rate of diabetic wound closure in the early stages of healing, thereby suggesting that they may have antiinflammatory properties.	Chouhan D et al., 2018 [23]
Nanofibers	Nanofibers carrying the bacterial inhibitor gentamicin sulfate (GS) and recombinant human epidermal growth factor (rhEGF)	*In vivo* model: Diabetic mice were given a wound of diameter 15mm. The wounds were then treated with one of the following: 1. Saline, 2. 0.1% GS solution, 3. Empty nanoscaffold, 4. Nanoscaffold with both GS and rhEGF or 5. A nanoscaffold with GS but without rhEGF.	The nanoscaffold containing both GS and rhEGF exhibited significantly greater wound closure after 4 days than all other treatments, although at 12 days this it was no better than the GS solution. The two active nanoscaffolds and the GS solution exhibited greater wound closure than saline and the empty scaffold throughout the investigation (p<0.01).	The rhEGF in the nanoscaffold is responsible for increased wound healing in the initial stages of the healing process.	Dwivedi C et al., 2018 [24]
Nanofibers	Poly(ether)urethane– polydimethylsiloxane / fibrinbased scaffold containing PLGA nanoparticles with themselves containing VEGF and FGF.	Diabetic mice were given wounds 8mm in diameter. The wounds were then treated with either saline, the active nanoparticle scaffold, an empty scaffold or a scaffold with growth factors but no nanoparticles.	Throughout the investigation there was no significant difference between the active nanoparticle scaffold and the growth factor scaffold, with the growth factor scaffold inducing slightly increased wound healing. However, at day 15 there was significant difference between these two groups and the two control groups (p<0.01).	The application of scaffolds containing growth factors to a diabetic wound can induce significant fibroblast proliferation and increased wound healing.	Losi P et al., 2013 [25]
Nanofibers	PLGA–collagen hybrid nanofibers containing rh-PDGF.	Diabetic rats were given wounds 5mm in diameter. The wounds were then treated with either a PLGA solution, a collagen solution or the active rh-PDGF nanoscaffold.	Throughout the 14 days of the investigation, the PDGF nanoscaffold significantly reduced the wound area when compared with the two control groups (p<0.05).	The PDGF nanoscaffold increases the rate of diabetic wound healing by increasing the amount of collagen in the wound.	Lee CH et al., 2016 [26]
Gold nanoparticles	Gold nanoparticles, epigal-locatechin gallate and alpha lipoic acid (AuNP+EGCG+ALA)	*In vivo* model: Diabetic mice were given wounds 1cm in diameter. Wounds were treated with either EGCG, ALA, EGCG & ALA or the AuNP composite. The wound area and the expression of proinflammatory factor RAGE (receptor for advanced glycation end products) were measured for 7 days after injury.	Wound area was significantly lower for the gold nanoparticle composite after 7 days (p<0.01). RAGE expression was also significantly lower for the gold nanoparticle composite after 7 days (p<0.01).	The gold nanopar-ticle composite significantly increases the rate of diabetic wound healing via antiinflammatory and angiogenic properties.	Chen SA et al., 2012 [27]
Gold nanoparticles	Gold nanoparticles, epigallocatechin gallate and alpha lipoic acid (AuNP+EGCG+ALA) – injected via a N2 gas carrier	*In vivo* model: Diabetic mice were given wounds 1cm in diameter. Wounds were treated with EGCG, ALA, AuNPs or AuNP+EGCG+ALA. The factors were applied to the wounds via a gas carrier each day for 7 days.	Wound area was significantly lower for the gold nanoparticle composite after 7 days until the end of the investigation (p<0.01).	The use of a gas carrier enhances the ability of AuNPs to produce collagen and hyaluronic acid, thereby increasing the rate of wound healing in diabetic subjects.	Huang YH et al., 2014 [28]
Gold Nanoparticles	Gold nanoparticles embedded in a silica (SiO2) matrix: (SiO2@AuNPs)	*In vitro* and *in vivo* models (the in vitro model showed that the AuNPs can induce the proliferation of mouse fibroblasts). In vivo model: Rats were given wounds of 2cm in diameter. The wounds were treated with either the gold nanoparticle matrix or a positive control of rh-FGF.	The level of hydroxyproline was higher in the rh-FGF between days 7 and 10, although by day 21, the gold NP matrix treated wounds contained significantly more hydroxyproline (p<0.05).	Gold nanoparticles embedded in a silica matrix can increase the proliferation of fibroblasts and therefore increase the production of collagen in the diabetic wound.	Li X et al., 2015 [29]
Liposomes	SDF-1 (Stromal cellderived Factor 1) embedded into liposomes.	*In vivo* model: Diabetic mice were given wounds 1cm by 1cm in size. The wounds were then treated with either 100µL of saline with 1µg SDF-1, 100µL of saline with 0.88µg of SDF-1 liposomes, 100µL of saline with 1µg of empty liposomes or 100µL of saline. The wound closure percentage (1 •remaining open wound area / initial wound area) was then calculated.	For the first 14 days and for day 28 there was no significant difference in wound closure between the active liposomes and the saline control. However, at day 21 the SDF-1 liposomes induced a significantly greater wound closure percentage (p<0.05).	In the later stages of wound healing, SDF-1 bound to liposomes may have a positive effect on wound closure. However, this link is not heavily substantiated.	Olekson MA et al., 2015 [30]
Liposomes	Epidermal growth factor (EGF), insulin-like growth factor-I (IGF-I), and platelet-derived growth factor-A (PDGF-A), all combined with protamine and hyaluronic acid and fused into liposomes.	*In vivo* model: Diabetic mice were given wounds 8mm in diameter. The wounds were then treated with either empty liposomes, liposomes with each of the growth factors at a concentration of 100µg /ml or liposomes with each of the growth factors at a concentration of 20mg / ml. The wound area was then measured for 11 days.	On days 1,3,9 and 11 the higher dose of growth factors significantly reduced wound area with p<0.001 and on day 7 the higher dose of growth factors significantly reduced wound area with p<0.01 when compared with the empty liposome control. After 11 days the lower dose of growth factors significantly reduced wound area when compared with the empty liposome control	The combination of several growth factors, alongside hyaluronic acid can significantly reduce wound area in diabetic patients by upregulating fibroblast proliferation and, therefore, collagen production.	Choi JU et al., 2017 [31]
